# On Glycerine and Its Therapeutic Uses

**Published:** 1849-12

**Authors:** 


*On Glycerine and its Therapeutic Uses.*—The employment of glycerine as a local application in the treatment of deafness, and also for other purposes as a medicinal agent, has induced us to offer a few remarks on the preparation and properties of this hitherto neglected body.

To obtain glycerine, any fatty matter is saponified by a caustic alkali. The solution being decomposed by tartaric acid, which precipitates the fatty acid, is to be evaporated, and the glycerine dissolved out by strong alcohol. For medicinal use, it is best obtained by evaporating the water used in making *emplastrum plumbi.* Any lead which it may contain is removed by a stream of sulphuretted hydrogen passed through it when in a diluted state. If necessary, it may be boiled with animal charcoal, filtered, and evaporated.

It is a colorless syrup, sp. gr.=1.26; it dissolves in water and alcohol, but is insoluble in ether (Kane's Chemistry, 1849, p. 872). Glycerine undergoes no change by keeping, but it is decomposed by heat; and when mixed with yeast, and kept in a warm place, is gradually decomposed, and converted into *metacetonic acid.* Its formula is $C_6H_7O_5+Aq$.

Glycerine has a strong attraction for moisture, and being of a bland, innocuous nature, it is obviously well adapted to afford a permanent, moist covering to any part of the body to which it may be applied. It was first employed by Mr. Yearsley in cases of deafness, arising from partial destruction of the membrane of the tympanum, and Mr. J. Wakley and others have extended its employment to all cases of deafness accompanied by dryness of the external meatus, and it is probable that in some cases of this kind, it may be more serviceable than the almond oil commonly employed to remedy a deficiency of cerumen. Dr. Paterson, of Edinburg, has obtained some improvement in one of three cases in which he applied

A small piece of cotton wool is moistened with the glycerine, and introduced into the ear. When there is loss or partial destruction of the tympanum, we are directed to use a very small quantity of wool, and to moisten it well with the glycerine. This is passed to the site of the membrane, and when there, 'the spot must be found which it is indispensable the wool should occupy and cover; for then only, and not till then, will success attend the application, and the patient regain the hearing.'

Glycerine has been employed also as a local application in psoriasis by Mr. Startin, and, it is said, with advantage.—*Monthly Retrospect*, Aug. 1849, from *Pharm. Journ.*, *Med. Times* and *Lancet.*

